# Anterior cingulate GABA and glutamate concentrations are associated with resting-state network connectivity

**DOI:** 10.1038/s41598-018-38078-1

**Published:** 2019-02-14

**Authors:** Nina Levar, Tessa J. Van Doesum, Damiaan Denys, Guido A. Van Wingen

**Affiliations:** 1 Amsterdam UMC, University of Amsterdam, Department of Psychiatry, Amsterdam Neuroscience, Amsterdam, The Netherlands; 2 Amsterdam Brain & Cognition, University of Amsterdam, Amsterdam, The Netherlands; 3 Spinoza Center for Neuroimaging, Amsterdam, The Netherlands

## Abstract

In recent years, resting-state (RS) networks and RS function have received increased attention, highlighting their importance in both cognitive function and psychopathology. The neurochemical substrates underlying RS networks and their interactions, however, have not yet been well established. Even though prior research has provided first evidence for a negative association between brain GABA levels and RS connectivity, these findings have been limited to within network connectivity, and not network interactions. In this multi-modal imaging study, we investigated the role of the main inhibitory neurotransmitter У-aminobutyric acid (GABA) and the main excitatory neurotransmitter glutamate (Glx) on RS network function and network coupling of three core networks: the default-mode network (DMN), salience network (SN), and central executive network (CEN). Resting-state functional connectivity and GABA and Glx levels in the dorsal anterior cingulate cortex (dACC) were assessed in 64 healthy male participants using functional MRI and magnetic resonance spectroscopy (MRS). Analyses showed that dACC GABA levels were positively correlated with resting-state connectivity in the CEN, and negatively associated with functional coupling of the DMN and CEN. In contrast, GABA/Glx ratios were inversely correlated with the SN and DMN. These findings extend insights into the role of GABA and Glx in individual networks to interactions across networks, suggesting that GABA levels in the SN might play a role in RS functional connectivity within the central executive network, and network interactions with the default-mode network. Our results further suggest a potentially critical role of the relationship between GABA and Glx in RS network function.

## Introduction

The human brain responds to both low and high demand cognitive tasks by changing its activity patterns across the brain. Depending on the nature of the cognitive demand, different brain networks engage and interact with each other during task performance. However, brain activity fluctuates across these task-specific activity networks even in the absence of any cognitive stimuli. In recent years, these so called resting-state (RS) networks have received increased attention, extending insights from task-based functional magnetic resonance imaging (fMRI) to brain function and brain networks that are active at rest^1^. These distinct networks are characterized by highly correlating activity patterns across different regions of the brain and have been shown to underlie various cognitive processes^2^. While neuroimaging research has provided ample insight into the brain structures underlying RS activity and networks, the underlying neurochemical substrates and their contributions to RS function have not been well established.

Recent advances using magnetic resonance spectroscopy (MRS) have provided initial insight into metabolite contributions underlying individual differences in RS activity across networks. Gamma-aminobutyric acid (GABA), the main inhibitory neurotransmitter in the human brain, has consistently been shown to be associated with blood-oxygen level dependent (BOLD) activity during both task-based and resting-state fMRI. While task induced BOLD activity has shown both positive and negative correlations with brain GABA levels^3–5^, depending on brain regions and cognitive processes, there is to date no clear understanding of the relation of GABA and RS network function and network interactions. Limited research focusing on the association between GABA and RS BOLD activity suggest a negative correlation, i.e. high GABA levels have been associated with decreased RS functional connectivity. However, studies have largely focused on the role of GABA and isolated regions within RS networks rather than investigating its role in RS connectivity across different networks and their interactions^6,7^. One study focusing on the DMN showed that GABA levels in the posterior cingulate cortex were inversely correlated with the strength of putamen and overall DMN connectivity^8^. Similarly, investigations of GABA levels within the posteromedial cortex (PMC) and intrinsic functional connectivity across the DMN revealed that GABA is negatively correlated with the strength of functional connectivity in the DMN^9^. In contrast to GABA, little is known about the role of glutamate, the main excitatory neurotransmitter in the brain, in resting-state network function. Prior research has focused on region-specific connectivity during task and resting-state fMRI, which revealed largely positive correlations^10–12^. The relationship between inhibitory and excitatory neurotransmitters has been suggested to play a similarly important role in brain function^13^, however, no studies have examined its role in RS network connectivity.

Even though these studies offer first insights into the relations of brain GABA and Glx levels and RS functional connectivity in the DMN, they do not examine how GABA and Glx affect activity in interconnected brain regions as a result of network interactions. A proposed triple network theory highlights three core networks which have been considered critical in a number of cognitive processes and psychopathologies: the salience network (SN), the default-mode network (DMN), and the central executive network (CEN)^14^. The SN comprises the fronto-insular cortex and the anterior cingulate cortex (ACC)^15^, and plays a key role in the communication and interplay between these three networks. The SN is responsible for determining and assigning saliency to events and stimuli^14^ and in turn initiates control on the CEN and the DMN, thereby regulating cognitive processes and function. The insula is considered to play a particularly important role within the SN by responding to behaviorally salient events and thereby serving as a critical hub for guiding (de)activation of the CEN and DMN. Specifically, when activation of the SN increases, activity in the central executive network increases as a result, while activation of the DMN is down regulated^14,16^. The DMN is involved in self-related cognitive functions and is comprised of the posterior cingulate cortex (PCC), precuneus, and the medial prefrontal cortex (mPFC)^17^. The core regions of the CEN include the dorsolateral prefrontal cortex (dlPFC) and the lateral posterior parietal cortex (PPC)^14,15^. This network is responsible for high-level executive function (e.g. working-memory and attention) and goal-directed behavior including decision making^18^.

Given the critical role of the SN within the triple network, SN GABA and Glx might play a particularly important role in RS network connectivity and in the communication between networks such as the DMN and the CEN. Task-based neuroimaging studies have highlighted the complexity of GABA-BOLD associations depending on task content and cognitive demand^3–5,19,20^, which might suggest a complex and differential role of GABA across and between networks also during rest. In this multi-modal MRI study, we applied magnetic resonance spectroscopy and functional MRI to investigate the role of GABA in the SN on RS network connectivity, by assessing the relationship between individual differences in SN GABA levels and RS connectivity (1) within the SN and (2) across DMN and CEN networks, and (3) how GABA impacts the role of the SN as a communication hub within the triple network. We expected GABA levels to negatively correlate with connectivity within the SN. We further hypothesized opposing directionalities of dACC GABA and network intraconnectivity of the DMN and CEN, and expected GABA to be associated with the interconnectivity between networks.

## Results

### Resting-state networks

**Figure 1:**
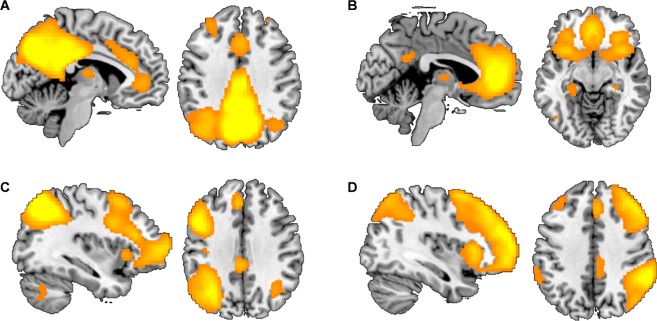
Resting-state networks identified using independent component analysis (ICA). Four independent components representing (**A**) the default-mode network (DMN), (**B**) the salience network, and the (**C**) left and (**D**) right central executive networks (CEN).

**Figure 2:**
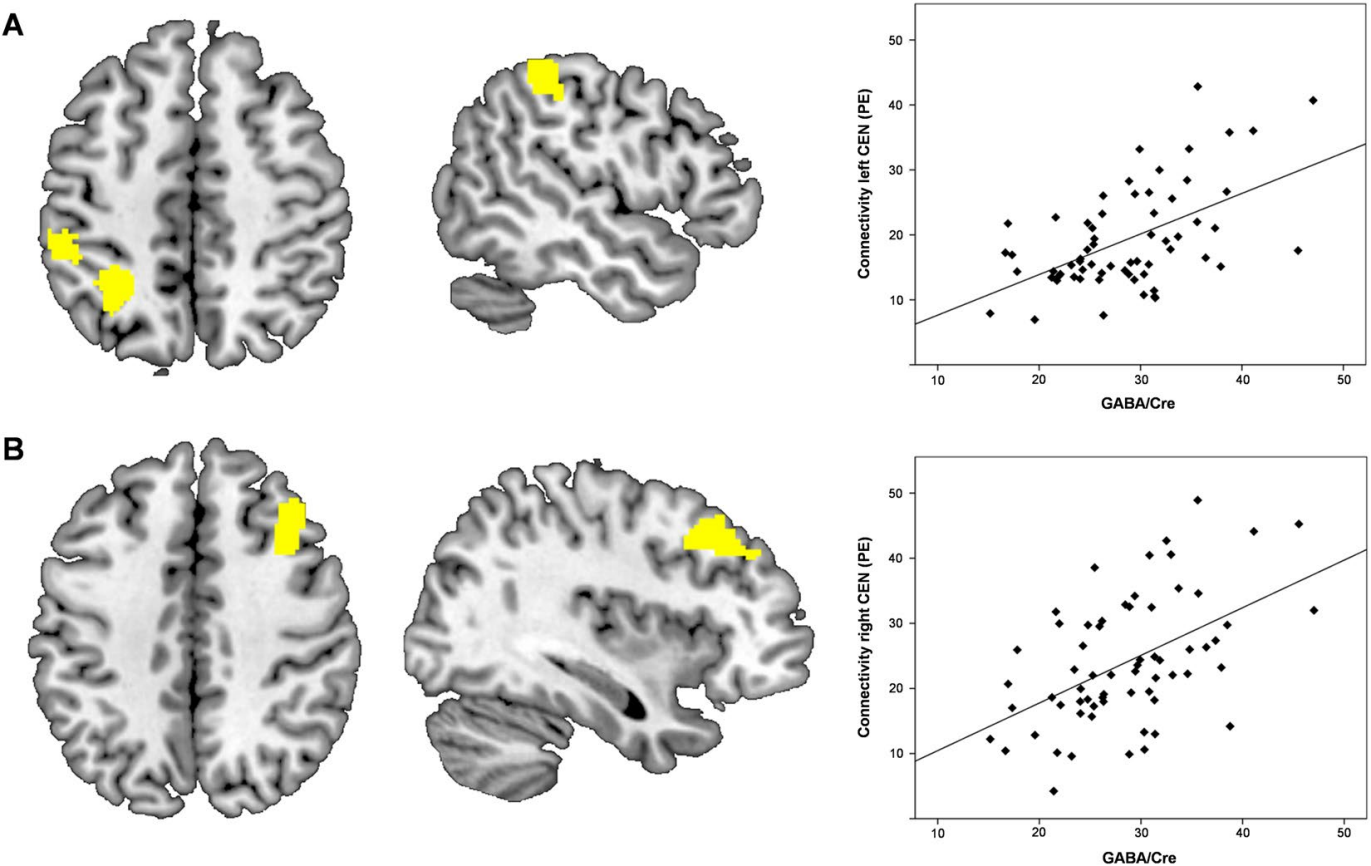
Correlations of dACC GABA and central executive networks. (**A**) Transverse and sagittal views of significant GABA associated functional connectivity (p < 0.05) in the lateral occipital cortex and superior parietal lobe of the left CEN. The scatter plot depicts the association between GABA/Cr levels and mean intensity (parameter estimates (PE)) of resting-state connectivity within the left CEN. (**B**) Transverse and sagittal views of significant GABA associated RS connectivity (p < 0.05) in the middle frontal gyrus of the right CEN. The scatter plot shows the correlation between dACC GABA/Cr and resting-state connectivity of the right CEN.

**Figure 3:**
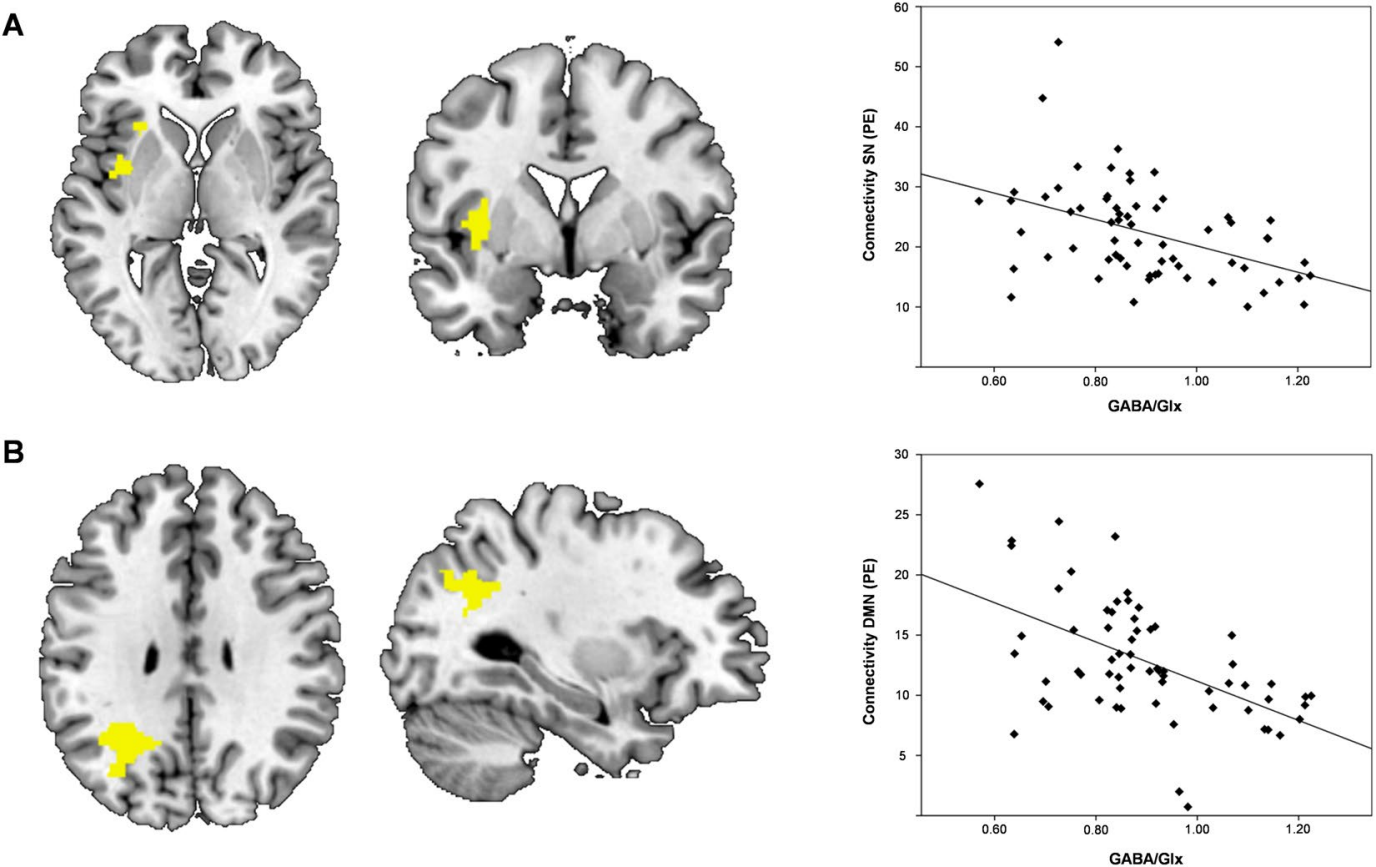
Correlations of dACC GABA/Glx ratios and the SN and DMN. (**A**) Transverse and sagittal views of significant GABA/Glx associated resting-state connectivity (p < 0.05) in the left insula of the SN. The scatter plot depicts the association between GABA/Cr levels and mean intensity (parameter estimates (PE)) of resting-state connectivity within the SN. (**B**) Transverse and sagittal views of significant GABA/Glx ratio associated functional connectivity (p < 0.05) in the lateral occipital cortex of the DMN. The scatter plot shows the correlation between dACC GABA/Cr and resting-state connectivity within the DMN.

Resting-state fMRI and GABA MRS data sets of 64 healthy right-handed male volunteers were acquired as part of a larger multi-modal imaging study. Resting-state data were analyzed using independent component analysis in Melodic in FSL (http://fsl.fmrib.ox.ac.uk/fsl/fslwiki/MELODIC). Salience, default mode, and left/right central executive networks were identified by visual inspection based on standard networks (Fig. 1)^2,14^. Left and right CENs were analyzed separately. The SN included the insula, dorsal ACC, and paracingulate gyrus. The DMN included the posterior cingulate cortex, the precuneus cortex, and the lateral occipital cortex (LOC). The left CEN included the left angular and supramarginal gyrus, inferior frontal gyrus, and the inferior temporal gyrus. The right CEN included the middle frontal gyrus, medial temporal gyrus, right frontal pole, the angular gyrus, and the supramarginal gyrus. We first assessed the association between dorsal ACC GABA levels and the SN. Metabolite and functional connectivity associations were determined by conducting a regression analysis using each participant’s GABA/Cr values as explanatory variables. This analysis revealed no significant correlations. Next, we assessed the association of dACC GABA with the CEN and DMN. GABA was positively correlated with RS connectivity in the superior parietal lobe (SPL) and LOC of the left CEN (Fig. 2). GABA was positively correlated with RS connectivity in the middle frontal gyrus of the right CEN. No region within the DMN showed any significant association with GABA levels in the dACC. Subsequently, we examined correlations between dACC Glx levels and RS functional connectivity. No significant correlations were observed for absolute Glx levels. Exploratory analyses assessing associations between GABA/Glx ratios and RS connectivity revealed significant negative correlations with the left insula of the SN and the LOC in the DMN (Fig. 3).

### Network Correlations

**Figure 4:**
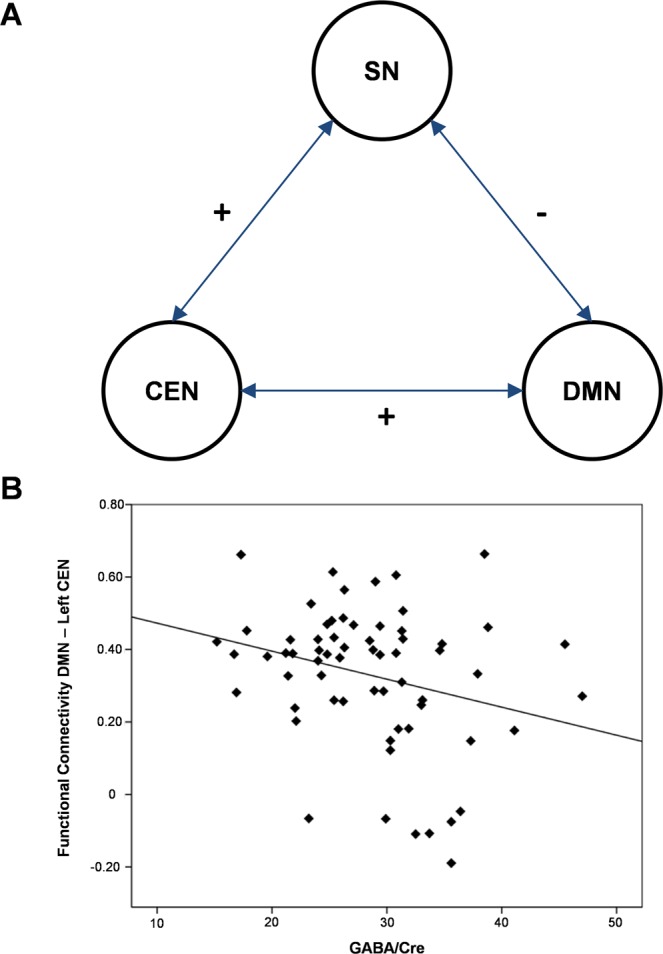
Resting-state network correlations. (**A**) Partial correlation analyses showed that the SN was positively correlated with the CEN and negatively correlated with DMN. CEN and DMN were positively correlated with each other. (**B**) Scatter plot showing that high GABA levels were correlated with reduced FC between the DMN and the left CEN.

A partial correlation analysis was used to assess unique contributions of each network within the triple network. Activation of the SN was negatively correlated with the DMN (r = −0.35, p < 0.001), and positively correlated with both the left and right CEN (left: r = 0.36, p < 0.001; right: r = 0.33, p < 0.001) (Fig. 4). The DMN was further positively correlated with the right CEN (r = 0.65; p < 0.001). Left and right CENs were furthermore positively correlated with each other (r = 0.25, p < 0.001). Correlation analyses with GABA levels in the dACC showed a negative correlation between GABA and each participant’s correlation coefficients of network interactions between the left CEN and DMN (r = −0.27, p = 0.03). No other significant associations were observed. No significant correlations were observed for Glx and GABA/Glx ratios.

## Discussion

The present study investigated the relation between GABA and Glx levels in the dACC, a core region of the SN, and individual differences in resting-state connectivity within the SN, and across the DMN and CEN in a group of healthy male participants. We further examined whether dACC GABA and Glx levels were associated with the interplay between these networks. This study revealed three main findings: (1) dorsal ACC GABA was positively correlated with RS connectivity in the left and right CEN, (2) dACC GABA/Glx levels were negatively correlated with RS connectivity in the SN and the DMN, and (3) dorsal ACC GABA levels were negatively correlated with network interactions between the left CEN and the DMN.

Even though the dACC is a core region of the SN, we did not observe the expected correlations between dACC GABA and connectivity within the dACC or other regions of the SN. There was, however, a significant positive correlation between GABA levels and functional connectivity in the LOC and SPL of the CEN, suggesting that individuals with high dACC GABA levels show increased activation of the CEN compared to individuals with low dACC GABA. These findings appear to be in contrast to previous studies which have largely provided evidence for a negative association between GABA and RS connectivity in DMN regions^8,9^. Our results therefore extend insights from prior DMN findings, indicating that SN GABA is associated with RS connectivity in distant regions rather than being correlated with activity directly within the SN and dACC. SN GABA might therefore play a role in guiding (de-)activation of the DMN, consequently resulting in an increase (or decrease, respectively) in CEN RS connectivity.

In contrast, we did not find any significant associations between dACC Glx and resting-state connectivity in any network, potentially suggesting that absolute SN Glx levels might not play a critical role in RS connectivity. Interestingly, the relationship between GABA and Glx was shown to be inversely associated with RS connectivity in both the SN and the DMN. This suggests that while absolute Glx levels might not be directly associated with RS connectivity, the relationship between GABA and Glx might play a critical role in RS function. Our data suggest that low GABA/Glx ratios are associated with increased connectivity within the SN and DMN, potentially highlighting differential roles of absolute and relative neurotransmitter levels in RS network function. With the SN playing a key role in guiding (de-)activation of the DMN and CEN, the excitatory/inhibitory balance might be critical in saliency detection and integration of information.

In addition to the relation of GABA and intra-network-connectivity, our results demonstrate that GABA was negatively associated with functional network coupling between the DMN and left CEN, suggesting that dACC GABA is not only associated with general resting-state connectivity of the CEN but might also affect interactions between the CEN and the DMN. This negative association between GABA levels and DMN-CEN coupling indicates that GABA levels in the SN may drive switching between the two networks, potentially affecting the shift between internal self-referential processes (DMN) to goal-directed resting state function (CEN). This is in line with the previously established role of the SN in detecting external salient stimuli and in turn, guiding interactions between internally oriented, self-related cognition (DMN) and externally oriented (CEN) goal-directed processes in response to these stimuli^14,16^.

Together, these findings highlight the importance of understanding neurotransmitter variations, particularly of GABA and Glx, and their relationship with cognitive function and RS connectivity in both healthy brain function as well as in psychopathology.^14,17,18,21–25^. This study provides a first step into revealing a potential role of dACC GABA and Glx in driving RS processes across different networks in a large sample of young adults, by combining both functional MRI and GABA magnetic resonance spectroscopy. Integrating our findings with previous research, this study provides contrasting evidence to the reported negative associations of brain GABA levels and RS connectivity. This could be explained by the use of different brain regions for MRS GABA measurements and/or smaller samples sizes in prior research studies. Furthermore, these studies have focused on regions within the DMN only, and have not examined its role across networks. Interestingly, recent advances in task-based MRS/fMRI studies have provided evidence for a more complex role of GABA on brain function, indicating both positive and negative associations of GABA and BOLD activity based on cognitive demand^3,4^. GABA might play a similarly opposing role in resting-state function, resulting in either positive or negative correlations depending on both brain region and type of RS network.

As a potential limitation to our findings, it is important to note that MRS provides us with a measure of total GABA metabolite concentrations, and it therefore remains unclear whether the GABA signal represents cytoplasmic, vesicular, or free extracellular GABA^26^. We further acquired GABA MRS data 24 hrs before the acquisition of resting-state data. Even though initial research in rodents has shown that GABA levels vary with the circadian rhythm^27^, more recent GABA MRS studies have suggested that GABA levels remain stable throughout and across days. Based on these findings, we assumed stability of GABA levels across the 24 hour time period^28–30^. Similarly, test-retest reliability of resting-state networks identified using independent component analysis and dual regression has been shown to be moderate to high across three scans acquired with a 5–16 months gap and a 45-minute delay^31^. Furthermore, this study investigated GABA levels in men only, because of the occurrence of hormonal fluctuations and varying GABA levels throughout the menstrual cycle^32^. Future research should extend these findings to women and examine the impact of hormonal fluctuations on GABA levels and RS connectivity.

Summarizing, GABA levels in the dACC of healthy male adults were positively correlated with RS network connectivity within the CEN, but not the SN, and showed a negative correlation with CEN-DMN coupling. These findings indicate that GABA levels within the SN influence CEN intra- and interconnectivity with the DMN, thereby potentially regulating network function and communication across networks. The present study extends insights from previously reported negative GABA-RS associations to a more complex role of GABA in RS function, revealing both positive and negative relations within networks and across distant network, respectively. Future research needs to assess whether these correlations are specific to the examined brain region and networks, and how these associations translate to other resting-state networks and cognitive processes.

## Material and Methods

### Participants

87 healthy right-handed male volunteers between 18 and 35 years (M = 21.75 years, SD = 3.17) participated in a resting-state paradigm as part of a larger two-day multi-modal imaging study. Other results of this study obtained during task-based fMRI have been reported previously^3,4^. Participants were screened to have normal or corrected-to-normal vision, no average use of more than three alcoholic beverages per day, no weekly use of recreational drugs, and no habitual smoking. Using self-report measures, it was further ensured that participants had no current or history of psychiatric, neurological, or endocrine disease, and recent irregular sleep/wake rhythms. Smoking and recreational drug usage was prohibited starting 24 and 72 hours, respectively, before entering the study until the end of the experiment. MRS data of 23 participants were either not acquired or excluded due to poor quality (see below). Complete data sets, GABA spectra and resting-state fMRI data, were available for 64 participants. Informed consent was obtained before entering the study. The study was performed in accordance with the declaration of Helsinki and approved by the Medical Ethics Committee of the Academic Medical Center in Amsterdam.

### MRI acquisition

**Figure 5:**
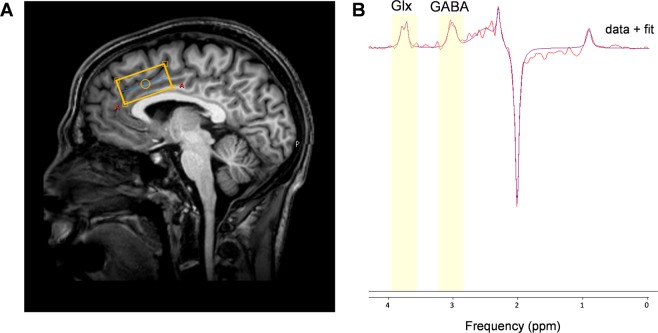
(**A**) MRS voxel placement in the dorsal anterior cingulate cortex. (**B**) MRS spectrum and fit. The GABA peak was fitted using a Gaussian function at 3.0 ppm. The Glx peak was fitted using two Lorentzian curves.

Neuroimaging data were acquired at the Spinoza Center (Amsterdam, The Netherlands) using a Philips Achieva 3T scanner using a 32-channel head coil. On day 1 of the experimental protocol, participants underwent a ^1^H MEGA-PRESS^33^ of the dACC. For accurate placement of the MRS voxel, high resolution structural images were acquired prior to the MRS scan using a protocol with the following parameters: TR/TE = 8.2 ms/3.8 ms, FOV = 240 × 188 mm^2^, voxel size = 1 × 1 × 1 mm^3^, 220 slices. Due to the experimental design, participants performed a five minute resting-state MRI scan approximately 24 hours after the MRS scan. Participants were instructed to keep their eyes closed throughout the resting-state scan. Whole brain echo planar images were acquired with the following scan parameters: TR/TE = 2000 ms/27.63 ms, slice gap = 0.3 mm, FOV = 240 × 240 mm^2^, voxel size = 3 × 3 × 3 mm³, and 37 slices. GABA-edited MRS spectra were acquired from a 40 × 20 × 20 mm³ volume place in the dorsal ACC using a MEGA-PRESS sequence^33^ with the following parameters: TR/TE = 2000/73, 384 averages, T_Acq_ = 12:48 min (Fig. 5). On all odd numbered acquisitions, selective inversion pulses were applied using 15.64 ms sinc-center pulses (64 Hz bandwidth) at the 1.91 ppm resonance of GABA. On even-numbered acquisitions, selective inversion pulses were applied symmetrically around the water resonance.

### fMRI analysis

We applied an independent component analysis (ICA) using Melodic in FSL (www.fmrib.ox.ac.uk/fsl/melodic2/index.html) to identify components representing the SN, DMN, and left and right CEN separately. Independent components were identified using a probabilistic approach to decompose BOLD data. A high pass filter cutoff of 1/150 Hz was used. Preprocessing included motion correction (MCFLIRT), slice time correction, BET brain extraction, and spatial smoothing with a FWHM kernel of 6 mm. Functional MRI images of each participant were registered to each individual’s structural image in standard MNI space. A linear registration was performed with a resampling resolution of 2 mm. Time courses were variance-normalized and multi-session temporal concatenation was performed as part of the analysis. The number of output components was restricted to twenty-five components. Best-fit components representing the salience, default-mode, and left and right central executive networks were selected from the resulting group components (Fig. 1). Using dual regression, time courses for each participant and for each independent component were extracted. Per network, a regression analysis was specified that included GABA/Cr and Glx/Cr values of each participant as explanatory variables. To identify regions with statistically significant correlations between network activity and GABA and Glx, we performed permutation tests (5000 interactions) and corrected for multiple comparisons at the cluster level (p < 0.05) using a cluster defining threshold of z > 2.3. To assess the interaction between networks and their unique contributions to network function, time courses for each participant and for each network of interest (SN, DMN, left + right CEN) were entered in a partial correlation analysis. To assess associations of GABA and Glx and network interactions, correlation coefficients of coupled time courses were extracted in order to allow for correlations with single value MRS metabolite concentrations. For each subject, we therefore entered correlation coefficients of network interactions in a correlation analysis with their respective GABA/Cr, Glx/Cr, an GABA/Glx concentrations and ratios, respectively.

### MRS analysis

GABA MRS data was pre-processed for final analyses using in-house software generating both even and difference spectra. The resulting spectra were analyzed using previously established procedures^3,4^ in AMARES in jMRUI^34^. Pre-processing of the spectra included apodization (Lorentzian 5 Hz), zero filling (1024), and hard-phasing of the signal around the N-acetylaspartate (NAA) peak. Difference spectra were phased to 180°. The GABA + (GABA + macromolecules) peak was fitted onto the difference spectra at 3.0 ppm using a single Gaussian function (phase = 0°). Even spectra were fitted for NAA (2.0 ppm), creatine (Cr) (3.0 ppm), and choline (Cho) (3.2 ppm) using a Lorentzian curve. Creatine was used as an internal standard for GABA + and Glx quantitation^35^. As spectra allowed for the quantitation of combined glutamate and glutamine (Glx), we determined Glx levels by fitting two Lorentzian curves onto the Glx peaks. GABA/Glx ratio were obtained accordingly. Spectra were excluded if the NAA peak width was larger than 11 Hz. GABA/Cr ratios were calculated for all participants and ranged from 0.15 to 0.47 with M = 0.29 and SD = 0.07. Glx/Cr ratios ranged from 0.19 to 0.56 with M = 0.33 and SD = 0.09. GABA/Glx ratios ranged from 0.57 to 1.22 with M = 0.90 and SD = 0.16. Glx/Cr ratios were square-root transformed to obtain a normal distribution for statistical analyses.

## Data Availability

The datasets generated during and/or analysed during the current study are available from the corresponding author on reasonable request.
